# Quality of life of individuals receiving kidney transplantation in Amazonas State

**DOI:** 10.1590/1518-8345.3775.3291

**Published:** 2020-06-08

**Authors:** Eliza Dayanne de Oliveira Cordeiro, Tatiana Castro da Costa, Monique Figueiredo Teixeira, Noeli das Neves Toledo, Gilsirene Scantelbury de Almeida

**Affiliations:** 1 Universidade Federal do Amazonas, Departamento de Saúde e Qualidade de Vida, Manaus, AM, Brazil.; 2 Prefeitura Municipal de Manaus, Secretaria Municipal de Saúde, Manaus, AM, Brazil.; 3 Universidade Federal do Amazonas, Subsistema Integrado de Atenção à Saúde do Servidor, Manaus, AM, Brazil.; 4 Universidade Federal do Amazonas, Escola de Enfermagem de Manaus, Manaus, AM, Brazil.

**Keywords:** Quality of Life, Renal Insufficiency, Chronic, Kidney Transplantation, Patient Care, Transplant Recipients, Public Health, Qualidade de Vida, Insuficiência Renal Crônica, Transplante de Rim, Assistência ao Paciente, Transplantados, Saúde Pública, Calidad de Vida, Insuficiencia Renal Crónica, Trasplante de Riñón, Atención al Paciente, Receptores de Trasplantes, Salud Pública

## Abstract

**Objective:**

to analyze the quality of life of kidney transplant receivers in the State of Amazonas.

**Method:**

a cross-sectional, descriptive study, performed with 222 individuals after renal transplantation registered in a private clinic and in a health public ambulatory. Data collection took place through structured interviews where the quality of life was measured by the Kidney Disease Quality of Life - Short Form. Descriptive statistics were used for data analysis.

**Results:**

the quality of life scores found ranged from 36.5 to 83.1. The quality of life domains, specifics of renal disease, have proved to be superior to generic ones. The most compromised were work situation; sleep; physical function and emotional function, with scores of 36.5; 53.7; 52.4; 55.1, respectively, and correlated moderately and significantly with each other.

**Conclusion:**

the majority (63.2%) of the quality of life domains obtained high scores and the specific component of renal disease had higher scores than the generic component.

## Abstract (pt)

**Objetivo:**

analisar a qualidade de vida de receptores de transplante renal no Estado do Amazonas.

**Método:**

estudo transversal, descritivo, realizado com 222 indivíduos pós-transplante renal cadastrados em uma clínica privada e em um ambulatório público de saúde. A coleta de dados deu-se por meio de entrevista estruturada onde a qualidade de vida foi medida pelo *Kidney Disease Quality of Life - Short Form*. Para a análise dos dados, foi utilizada estatística descritiva.

**Resultados:**

os escores de qualidade de vida encontrados variaram de 36,5 a 83,1. Os domínios de qualidade de vida específicos da doença renal mostraram-se superiores aos genéricos. Os mais comprometidos foram Situação de trabalho; Sono; Função física e Função emocional, com escores 36,5; 53,7; 52,4; 55,1, respectivamente, e correlacionados moderada e significativamente entre si.

**Conclusão:**

a maioria (63,2%) dos domínios de qualidade de vida obtiveram escores elevados e o componente específico da doença renal teve pontuações superiores ao componente genérico.

## Abstract (es)

**Objetivo:**

analizar la calidad de vida de las personas receptoras de trasplante renal en el Estado de Amazonas.

**Método:**

estudio transversal descriptivo, realizado con 222 personas, con posterioridad a la realización de un trasplante renal, inscriptos en una clínica privada y en un hospital público. La recolección de datos se realizó mediante entrevista semiestructurada y se midió la calidad de vida por medio del *Kidney Disease Quality of Life - Short Form*. En el análisis de datos, se utilizó la estadística descriptiva.

**Resultados:**

El puntaje de calidad de vida obtenido osciló de 36,5 a 83,1. Los dominios de calidad de vida propios de la enfermedad renal, tuvieron un puntaje superior al genérico. Los más comprometidos fueron: situación laboral, sueño, función física y función emocional, con puntajes de 36,5; 53,7; 52,4; 55,1, respectivamente, y correlacionados moderada y significativamente entre sí.

**Conclusión:**

la mayoría (63,2%) de los dominios relativos a la calidad de vida obtuvieron puntajes elevados y el componente específico de la enfermedad renal obtuvo un puntaje superior al componente genérico.

## Introduction

The worldwide prevalence of end-stage chronic kidney disease (CKD) has increased considerably in recent years. The technological advances related to Renal Replacement Therapies (RRT) - hemodialysis (HD), peritoneal dialysis (PD) and renal transplantation (Tx) have made possible greater survival for patients, even with some sequels and/or comorbidities^(^ 1 ^)^. In this scenario, Brazil stands out in the coordination and regulation of organ transplants in public services, ranking 2^nd^ in absolute number of transplants in the world^(^ 2 ^)^, but it falls to 25^th^ place when the proportion of its population is considered^(^ 3 ^)^.

It is important to emphasize that renal Tx has already been characterized as the best alternative, financially and clinically, in cases of end-stage CKD, generating significant savings to the State coffers^(^ 4 ^)^. Despite this, Brazil has remained with the renal Tx rate unchanged for the last six years: 28.5 pmp and less than 50% of the estimated need for kidney Tx was actually accomplished in the last year. There is also a drop (32.9%) in the rate of transplantations with a living donor in contrast to the small increase (10.3%) in the rate with a deceased donor^(^ 3 ^)^.

Although kidney failure has gained importance in the list of chronic diseases, there is a reduced number of articles concerning the subject. Among them, those referring to hemodialysis users predominate, highlighting the health dimensions related to CKD and how they influence the quality of life (QL)^(^ 5 ^)^.

To evaluate the effectiveness and even assist the choice among the available treatment modalities, quality of life has often been used as a parameter around the world^(^ 6 ^)^. For this purpose, studies have been presenting instruments designed to measure QL generically and also specifically in groups of individuals with different characteristics of the population in general, such as those with CKD.

One of the most famous and currently used generic QL instruments is the Health Survey SF-36, published in 1993, translated and validated in Brazil in 1997. From it, was the Kidney Disease Quality of Life - Short Form (KDQOL-SF) derived, published in 1997 and specific for the measurement of QL in chronic renal patients under dialysis treatment. In 2003, KDQOL-SF was translated and validated for Brazil and today is one of the most complete QL instruments available for the renal-ill population^(^ 7 ^)^.

In 2006, a study in Budapest/Hungary demonstrated that KDQOL-SF is also reliable and valid for post-Kidney Transplantation patients, and can even be used to compare different populations with end-stage kidney disease^(^ 8 ^)^. However, few studies have been published that analyze the quality of life of kidney transplant receivers through KDQOL and only one of them is Brazilian^(^ 6 ^)^.

This single national study was carried out in the Southern region fo the country that, which in turn has discrepancies with the northern region as regards population, geography, culture, development, economy, access to health goods and services, in addition to the differences in the transplant service which place the two regions at opposite ends with regard to the kidney transplant rate, with the southern region standing at 1^st^ place, with a rate 11.16 times higher than that of the North, last place in the country^(^ 3 ^)^.

This is also due to the fact that three of the seven states that make up the Northern region did not perform kidney transplants in the last year (Amapá, Roraima, and Tocantins) and the State of Amazonas performed only one kidney Tx^(^ 3 ^)^. These considerations reinforce the existing differences between the regions of Brazil and the need for a study on the subject in the North and in other regions of the country.

The aim of this study was to analyze the quality of life of kidney transplantation receivers in the State of Amazonas.

## Method

This is a descriptive, cross-sectional, quantitative approach study that was carried out in the only two institutions that follow post-renal transplantation patients in the State of Amazonas, one public and the other private, both located in the capital city of Manaus.

Prior to the start of the collection, the institutions provided a letter of consent to conduct the survey and a list with the post-Tx patients registered in each of them: 194 users in the public outpatient clinic and 74 in the private clinic, totaling 268 transplanted individuals that correspond to the study population.

For patient selection, the following criteria were followed: be of legal age (≥18 years); be based in the State of Amazonas; be available in Manaus/AM during the collection period; to have undergone the transplantation at least three months ago; have the renal graft working and/or not undergoing dialysis therapy and have cognitive conditions to respond to the instruments. Besides, four transplanted patients were excluded because they declared themselves to be indigenous, since there was no viable time to obtain the consent of the organs specifically responsible for conducting research with this population. Thus, 222 (82.8%) kidney transplanted individuals composed the final sample.

Data collection took place from June 2018 to February 2019. During this period, an active search for all transplanted members of the two provided lists was carried out, by means of telephone calls, user associations, virtual social networks, in addition to being invited in person at the moment they came for the follow-up consultation in the institutions. Those who could not be reached or did not attend the post-Tx return consultations and/or could not be contacted after three or more telephone attempts were considered “non-localized”.

During the data collection, individual interviews were conducted in a reserved place previously agreed upon with each user, usually in the outpatient or clinic itself. Each transplanted individual responded to a population profiling form and the KDQOL-SF.

KDQOL-SF consists of 21 QL subscales subdivided into 80 items, including the Medical Outcomes Survey - 36 Item Short -Form Health Survey (SF-36) as a generic measure, in addition to the items specific to the chronic renal patient particularities^(^ 7 ^)^. KDQOL-SF is of public domain, available at the Research and Development Corporation (RAND)^(^ 9 ^)^ website, the developer, and composed of 19 dimensions of QL, eight of them coming from SF-36: Physical function; Emotional function; Social function; Emotional well-being; Pain, Energy/Fatigue; General health and a item on current Health computed separately. Eleven, in turn, are from the specific part of KDQOL-SF: Symptoms/Problems; Effects of kidney disease; Overload of kidney disease; Work situation; Cognitive function; Quality of social interactions; Sexual function; Sleep; Social support; Stimulation of the dialysis team; Patient satisfaction and a general health item computed separately^(^ 7 ^)^. In this study, five items corresponding to two dimensions of the specific part of the instrument were excluded because they were aimed at dialysis patients.

KDQOL-SF’s responses yield scores from zero to 100, where higher values always represent better quality of life states. Each dimension of QL corresponds to the arithmetic mean of the items that compose it. The manual with the coding and interpreting instructions is available on the *RAND* website^(^ 9 ^)^.

For data analysis, descriptive statistics were used: absolute and relative frequencies for questions regarding the characterization of the study population and means and standard deviations for the quality of life dimension scores. Subsequently, Pearson’s correlation test was used between scores of the most compromised QL dimensions, where values of *r* between 0.1 and 0.29 were considered as a small effect; between 0.3 and 0.49, mean effect, and higher or equal to 0.5, a large effect. To evaluate the reliability and internal consistency of the instrument responses, Cronbach’s alpha coefficient (α) was calculated, where values higher than 0.7 are considered reliable. The collected data was stored, coded, analyzed and interpreted using the Statistical Package for Social Sciences SPSS^®^ for Windows^®^, version 21.0.

The national ethical recommendations on research with human beings, recommended by the National Health Council, were met, and the study was approved by the research ethics committee of the Federal University of Amazonas under opinion no. 2,724,945 and CAAE 88684218,7,0000,5020.

## Results

The final sample of the study consisted of 222 kidney transplanted individuals, among whom men predominated (60.4%), married (45.5%), mean age 45.8 (±12.8) years, brown (74.3%), with 11.3 (±4.6) years of study. As for the municipality of current residence, 194 (87.4%) lived in Manaus (capital). The public service was responsible for monitoring 77% of transplanted individuals. Regarding the type of organ donor, 110 received from family donors, 35 from living donors, not relatives, and 77 (34.7%) from deceased ones. The majority (72.1%) denied current work, but personal income varied from 0.0 to 30.1 minimum wages, with a median of 1.3 [1.0;3.3].

The results regarding the health-related QL of the study group are shown in Table 1 and divided between the scores of the generic and specific health domains of CKD. The Cronbach’s alpha coefficient was equal to 0.95, indicating a strong internal consistency and reliability of the responses found.

**Table 1:** Descriptive analysis of quality of life scores (KDQOL-SF*) in kidney transplant receivers in each of their domains. Manaus, AM, Brazil, 2018-2019 (n=222)

Quality of life domains	Mean(±DP^‡^)
KDQOL*	
Symptoms/problems	83.1 (±14.5)
Effects of kidney disease	83.1 (±16.3)
Kidney disease overload	71.0 (±28.8)
Work situation	36.5 (±40.0)
Cognitive function	82.8 (±19.3)
Quality of social interaction	77.7 (±20.7)
Sexual function	75.4 (±25.4)
Sleep	53.7 (±17.3)
Social support	83.5 (±23.0)
General health assessment	78.5 (±16.8)
**SF-36^†^**	
Physical functioning	73.1 (±26.1)
Physical function	52.4 (±39.7)
Emotional function	55.1 (±43.5)
Social role	75.7 (±27.1)
Emotional wellbeing	73.7 (±21.2)
Pain	78.7 (±25.2)
Vitality (Energy/Fatigue)	67.1 (±21.1)
General health	64.7 (±24.3)
Current health - compared to one year ago	61.9 (±25.7)

* KDQOL-SF = Kidney Disease Quality of Life;

† Short Form;

‡ SD = standard deviation

Table 1 shows that Sleep (53.7) and Work Situation (36.5) had the lowest means among the specific dimensions of renal disease, with Work Situation (or Professional Role) being the lowest mean (36.5) among them. As far as the generic dimensions of health are concerned, none of them have shown such low value. However, among them, the lowest means were in Physical Function (52.4) and Emotional Function (55.1).

By analyzing the relationship between the most compromised dimensions of the specific (Sleep and Work) and generic (Emotional Function and Physical Function) components of KDQOL, by Pearson’s correlation test, moderate correlations are found between Sleep and Emotional Function (r=0.32) and Work and Physical Function (r=0.42), both significant at level 0.01.

## Discussion

This is the first study to analyze the quality of life of kidney transplant receivers in the State of Amazonas, being also the second Brazilian to use a specific QL tool for patients with chronic kidney disease in this type of individual.

We observed a population similar to that found in other national^(^ 6 ^,^ 10 ^-^ 11 ^)^ and international^(^ 8 ^,^ 12 ^)^ studies with regard to sex, age, marital status, place of residence (capital/upstate), work and complete years of study.

As for the color/race self-reported, few studies on the subject have disclosed this characteristic of the population and, among them, the white color/race was predominant^(^ 11 ^,^ 13 ^)^, opposing the great majority of brown individuals found in this research. These observations are in line with the Brazilian scenario where the majority of the population declares itself white, mainly in the South (78.5%) and Southeast (55.2%) regions (where the largest number of scientific publications come from), as opposed to the other regions, such as the North, where the majority of the population declares itself brown (66.9%)^(^ 14 ^)^.

As for the type of donor, the higher number of living donors found in the survey opposes the national scenario, where the number of transplants with deceased donors has always been higher. In 2018, only 17.18% of the renal Tx in the country were from living donors^(^ 3 ^)^. A similar study conducted in a municipality in the southern region of the country also found a prevalence of renal Tx coming from living donors (75%)^(^ 6 ^)^. The type of donor most frequent in Amazonas is also a reflex of the actual organ donors’ rate: 3 pmp, well below the national rate of 17 pmp^(^ 3 ^)^.

It should be noted here that the transplant by deceased donor only started to take place in Amazonas in 2011. Even so, the logistics of transportation to this state makes it difficult to receive organs from other regions in time for the surgery. In the case of the kidney, it needs to be grafted within 24 hours to ensure the possibility of successful transplantation.

We stress the need for attention and improvement in the attraction of living donors (potential and effective), a problem that is not restricted to the locality or country, but rather to transplant programs around the world.

A study conducted in Andalusia, an autonomous region of Spain, where 45% of the kidney receivers from a living donor declared that they needed more information about this type of Tx. We also add in this study that only 7.5% of these receivers received information on Tx from a living donor by a nurse. With regard to donors, none reported being informed on the subject by this professional^(^ 15 ^)^, highlighting the importance of wider dissemination of information on the kidney donation process and greater awareness of people in order to create greater opportunities to attract potential living donors.

In general, observing the results of Table 1, it can be seen that the scores of the instrument’s specific domains were better than those of the generic domains. Even so, it was also in the specific part that the two dimensions with the worst KDQOL scores were found, demonstrating the importance of using instruments that take into account the specificities of a group with differentiated characteristics such as CKD carriers.

When comparing the QL results of other studies with those presented here, it was found that, in Amazonas, the post-Kidney Tx patient’s QL is lower, in most dimensions, than the receivers in Rio Grande do Sul^(^ 6 ^)^, state located in the South region of the country, which, in turn, is more developed and with much better rates and evaluations than those of the North region regarding the national transplant system.

In relation to other countries around the world, the transplanted individuals in this study had higher scores than most countries that published KDQOL-SF results in this type of population, such as Norway, Hungary, Poland, United States of America (USA), among others^(^ 8 ^,^ 16 ^-^ 18 ^)^, which may be a reflection of the Brazilian Public Health System (SUS), based on a comprehensive concept of universality, which provides coverage and comprehensive health care for the entire population, not excluding, of course, other possible related factors^(^ 19 ^)^.

Regarding the labor situation, the majority of transplanted individuals (72.1%) said they were not working, but the median of personal income was 1.3. This counterpoint is because most of them claim to receive sick pay or disability retirement. Also, some have reported informal means of income composition, a reality consistent with the literature, since informal jobs are more easily adaptable, and kidney-transplanted can choose the best activity that suits their capabilities, general condition, schedules and more flexible periods^(^ 20 ^)^.

This aspect directly reflected in the “Work situation” dimension that ended up presenting the lowest score (36.5±40) among transplanted patients. This dimension was also one of the most compromised in similar studies with post-Kidney Tx patients in Brazil^(^ 6 ^)^ and in other countries^(^ 16 ^-^ 17 ^,^ 20 ^)^.

Another specific domain of KDQOL-SF that was quite compromised was Sleep (53.7±17.3), with a score very below that of a similar study carried out in the South region of Brazil, with a mean equal to 77.4 (±19.5)^(^ 6 ^)^. In international studies that used the same instrument after kidney transplantation, the lowest score found in the Sleep domain was in Boston/Massachusetts in the USA, in 2016^(^ 18 ^)^, with 64,1(±16.7) of mean.

As for the generic part of the instrument, derived from the SF-36, the most compromised dimensions were Physical Function (52.4) and Emotional Function (55.1), which refer to the point at which physical and emotional issues interfered with an individual’s daily activities or functions and whether they have needed to reduce these activities because of these issues. In another Brazilian study, conducted in a city in the South of the country, the Physical Function and the Emotional Function obtained scores 11.1% and 15.7% higher than those in the Amazonas^(^ 6 ^)^. No other generic QL dimension of the study conducted there had scored so low as those found here.

Among similar international studies, some also had the dimension Physical Function among those with lower scores^(^ 8 ^,^ 17 ^,^ 21 ^)^, however, the Emotional Function got good scores in these studies. Only two studies, one conducted in Warsaw/Poland and the other in Daka/Bangladesh, had such a compromised Emotional Function, lower than the study presented here, with scores of 37.33 (±43.6) and 46 (±44), respectively^(^ 17 ^,^ 22 ^)^.

As mentioned above, there was a moderate and significant correlation between the dimensions Sleep andEmotional Function, and Work SituationandPhysical Function. This correlation may have been reinforced by the precarious post-Tx follow-up service at the time of collection, by prioritizing specialized medical evaluation over multidisciplinary follow-up, evident in the private network but also committed in the public network.

The results found here point out to the need for health education on the care measurements and lifestyle post-renal Tx, including orientations and the promotion of activities aimed at the practice of physical exercise allowed for this public. In this regard, Nursing can be a key point for interventions, since it is the professional who still has the most frequent and prolonged contact with clients in post-Tx outpatient treatment.

In addition, we suggest special attention from the health professionals involved in what concerns the quality of the transplanted patients’ sleep, its related factors and sleep regulation mechanisms or strategies. It is also necessary to raise awareness among the population about transplantation and campaigns to promote organ donation, making public studies that show the improvement in QL, both of the receiver and the donor^(^ 22 ^)^, as well as investments in the service of attracting local organs and in performing transplants.

A limitation found in this study concerns the cross-sectional cut, which hinders the determination of cause-effect relationships between the variables studied. Moreover, the quantitative method does not cover the totality and depth of the multifactorial and complex issue of quality of life in health.

Therefore, it is suggested the development of future studies with differentiated approaches that can encompass the complexity and multifactorial aspects that is quality of life in health, among them: longitudinal, that can demonstrate the evolution and modifications of QL over the post-Tx years; that investigates the psychological/psychiatric commitment of this clientele; that seek to analyze the areas indicated here as compromised (physical, emotional/mental and work) and qualitative, allowing to deepen into the capture of the feelings and perceptions of the individual who lives with CKD over the years, including those who have achieved the best therapeutic alternative in cases of end-stage CKD; addressing their expectations, fears, losses, victories and current and future prospects.

## Conclusion

Health QL self-reported through the Kidney Disease Quality of Life - Short Form had better means in the specific component of the instrument, which proved to be consistent and reliable for the study’s renal transplanted patients. The QL dimensions most compromised, according to KDQOL-SF, were “Work Situation”, “Physical Function”, “Sleep” and “Emotional Function”, which have been correlated in a moderate and meaningful way with each other.

Kidney transplant receivers from the North region of Brazil have a lower quality of life than those from the South region of the country, which in turn is more developed and with better rates and evaluations regarding the national transplant system. Compared to other countries that published similar studies, predominantly in Europe, the QL scores of Brazilian kidney transplanted patients were higher, which may be a reflection of the Unified Health System in the country, free and available to all Brazilian citizens, not excluding, of course, other possible related factors.
